# Glomerular tip adhesions predict the progression of IgA nephropathy

**DOI:** 10.1186/1471-2369-14-272

**Published:** 2013-12-05

**Authors:** Kunihiro Maeda, Shogo Kikuchi, Naoto Miura, Keisuke Suzuki, Wataru Kitagawa, Hiroyuki Morita, Shogo Banno, Hirokazu Imai

**Affiliations:** 1 Division of Nephrology and Rheumatology, Department of Internal Medicine, Aichi Medical University School of Medicine, Aichi 480-1195, Japan; 2 Department of Public Health, Aichi Medical University School of Medicine, Aichi 480-1195, Japan

## Abstract

**Background:**

Focal segmental glomerulosclerosis-like lesions have been proposed to be predictive factors for IgA nephropathy. This single center, retrospective cohort study was designed to clarify which clinical and pathological factors are predictive of decreased estimated glomerular filtration rate (eGFR) at 5 and 10 years in IgA nephropathy patients.

**Methods:**

Of the 229 patients with IgA nephropathy who were admitted to Aichi Medical University Hospital between 1986 and 2010, 57 were included in this study during the 5 to 10 years after renal biopsy. Clinical, laboratory, and pathological parameters were analyzed by multiple linear regression analysis with backward elimination to determine independent risk factors. After identifying such factors, we compared patients with and without each factor using the Student’s t test, Wilcoxon test, or Mann–Whitney U test.

**Results:**

Four variables were identified as predictive factors for progression of IgA nephropathy: initial eGFR (p = 0.0002), glomerular tip adhesion (p = 0.004), global sclerosis (p = 0.019), and diastolic blood pressure (p = 0.024). The annual decrease in eGFR of patients with (n = 9) or without glomerular tip adhesions (n = 48) was 4.13 ± 3.58 and 1.49 ± 2.89 ml/min/1.73 m2, respectively (p = 0.015). Serum total cholesterol levels were 231 ± 45 mg/dl and 196 ± 42 mg/dl, respectively (two-sided p = 0.064; one-sided p = 0.032).

**Conclusions:**

The presence of glomerular tip adhesions predicts the progression of IgA nephropathy. High levels of serum total cholesterol may affect glomerular tip adhesions.

## Background

IgA nephropathy is the most common glomerulonephritis, defined as predominantly IgA deposition in the mesangial area with mesangial proliferation. It varies from mild focal segmental proliferation to severe diffuse global proliferation with crescent formation. Many studies examining the relationship between the histopathological features of IgA nephropathy and clinical outcome suggest that glomerulosclerosis and interstitial fibrosis are predictive of poor prognosis [1]. Mesangial hypercellularity [2,3], crescent formation [4-6], capillary wall IgA deposition [4,7], and focal segmental glomerulosclerosis (FSGS)-like lesions [8,9] have been suggested as histological risk factors for progressive renal failure in IgA nephropathy. Recently, FSGS-like lesions [10] and collapsing and cellular types of FSGS [11] have been proposed as predictive factors. These apparently conflicting results reflect differences in patient cohort, treatment, and clinical outcome measures. When the clinical end point is time to dialysis or renal failure, or serum creatinine doubling time, chronic lesions such as tubular atrophy, interstitial fibrosis, and glomerulosclerosis are identified as risk factors. If a decrease in the glomerular filtration rate (GFR) in early-stage disease is the chosen end point, other predictive factors may be identified. Idiopathic FSGS is classified into several subtypes such as the glomerular tip variant, cellular variant, collapsing variant, perihilar variant, and not otherwise specified (NOS) [12]. Within glomerular tip lesions, there are glomerular tip prolapse, glomerular tip adhesion, glomerular tip sclerosis, and glomerular tip foam cells. There are no data on which histological characteristics of FSGS are relevant to the progression of IgA nephropathy, although one study suggested the importance of the collapsing and cellular variants [11].

We designed a retrospective, single center cohort study to clarify which clinical and pathological factors are predictive of decreased eGFR in patients with IgA nephropathy at 5 and 10 years after renal biopsy, which includes an analysis of many pathological findings related to FSGS-like lesions.

## Methods

The present retrospective cohort study was approved by the Ethics Committee of Aichi Medical University.

**Figure 1:**
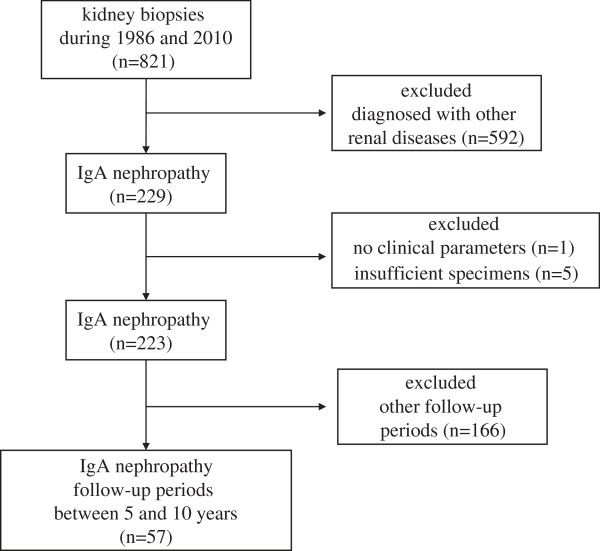
**Flow diagram in this study.** Flow diagram illustrates the selection of biopsy-proven IgA nephropathy patients followed for 5 to10 years that were included in this study.

Of the 821 patients who underwent renal biopsy between 1986 and 2010 at Aichi Medical University Hospital, 229 patients (27.9%) were diagnosed with IgA nephropathy based on the light microscopic and immunofluorescent study findings. In this group, 57 had sufficient clinical, laboratory, and pathological data and 5 to 10 years of follow-up (Figure 1).

### Analysis of clinical and laboratory data

We collected clinical and laboratory data from medical charts or computer-based data storage in the hospital, including the age at renal biopsy, sex, systolic blood pressure, diastolic blood pressure, dairy amount of urinary protein, degree of hematuria, total protein, serum albumin, blood urea nitrogen, creatinine, total cholesterol, initial estimated glomerular filtration rate (eGFR), and final eGFR, which were calculated using the 4-variable Modification of Diet in Renal Disease (MDRD) Study equation [13]. ΔeGFR was defined as initial eGFR minus final eGFR. The annual decrease in eGFR was calculated as (365 days) times ΔeGFR divided by the number of observation days.

### Analysis of pathological findings

**Figure 2:**
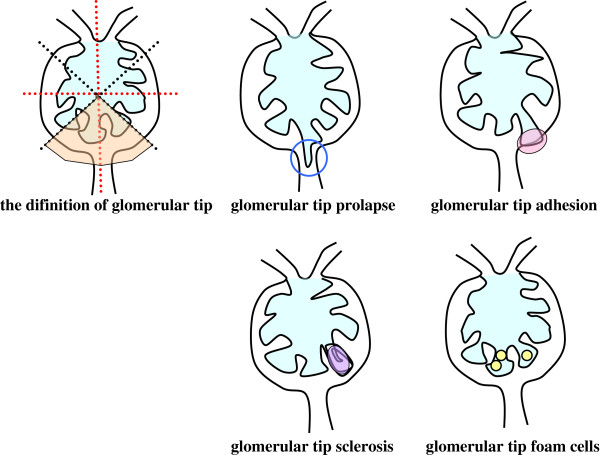
**Definition of glomerular tip and types of glomerular tip lesions.** The glomerular tip is defined as the outer 25% of the tuft next to the origin of the proximal tubule, based on the Columbia Classification. Glomerular tip prolapse means that some component of the glomerulus, such as the basement membrane or the mesangial cells, is prolapsing into the proximal tubules. Glomerular tip adhesion is defined as a continuity of the extracellular matrix between the glomerular tuft and Bowman’s capsule in the tip domain. Glomerular tip sclerosis is defined as segmental sclerosis in a portion of the glomerular tip. Glomerular tip foam cells refer to the presence of foam cells in a portion of the glomerular tip.

Four observers (KM, NM, HM, HI) independently evaluated the biopsy specimens under light microscopy with hematoxylin and eosin (HE), periodic acid-Schiff (PAS), periodic acid-silver methenamine (PAMS), and Masson’s trichrome (MT) staining. All samples contained more than 8 glomeruli, and 8 to 10 sliced sections per patient were observed. The pathological data included the total number of glomeruli, number of global sclerotic glomeruli, number of intact glomeruli remaining, number of glomeruli with crescent formation, total number of glomeruli with adhesions (i.e., the number of glomerular tip adhesions plus the number of non-glomerular tip adhesions), degree of mesangial proliferation, and degree of interstitial damage. In order to focus on FSGS-like lesions, the glomerular tip domain is defined as the outer 25% of the tuft next to the origin of the proximal tubule, based on the Columbia Classification [12]. In this classification of FSGS, glomerular tip lesions consist of tip prolapse, tip adhesion, tip sclerosis, and intracapillary foam cells in the tip domain (Figure 2). Glomerular tip adhesion was defined as continuity of the extracellular matrix between the glomerular tuft and Bowman’s capsule in the tip domain, [12] which is often thickened.

Tubular atrophy and interstitial fibrosis were assessed according to the criteria of El Karoui et al [10]. By light microscopy, the severity of tubular atrophy, interstitial cellular infiltration, and interstitial fibrosis, whichever the greatest, was semiquantitatively scored on a scale of 0 to 4 (0, none; 1, 0–25%; 2, 26–50%; 3, 51–75%, and 4, >75%).

### Statistical analysis

A stepwise logistic regression model was used with each variable and ΔeGFR/days as the outcome variable. All analyses were performed with SAS software version 9.1 (SAS Institute, Inc., Cary, NC, USA). Receiver operating characteristic (ROC) curve analysis was used to determine the cut-off point for variables that showed significant difference.

Subsequently, we compared two groups with or without one factor using the chi-square test. Quantitative values are expressed as means ± SD, unless otherwise noted. Comparisons were performed using the Student’s t test, Wilcoxon test, or Mann–Whitney U test as appropriate. P values less than 0.05 were considered statistically significant.

## Results

### Determination of factors influencing the decrease in eGFR

The backward elimination method was used to determine factors that strongly influence the decrease in eGFR. Briefly, the F value was determined for age, blood pressure, total protein, albumin, serum creatinine, eGFR, total cholesterol, and daily amount of urinary protein at the time of renal biopsy, number of globally sclerotic glomeruli, number of glomeruli with crescent formation, glomerular adhesion, or glomerular tip adhesion, which were divided by the number of glomeruli observed, and the tubulointerstitial damage score as defined in the Methods. Factors with the smallest F value were eliminated one by one. As a result, 6 factors remained: serum albumin, diastolic blood pressure, eGFR, number of globally sclerotic glomeruli, glomerular adhesion, and glomerular tip adhesion / the number of glomeruli observed.

Based on the multiple linear regression analysis, p values for these 6 factors were determined. The p value of eGFR at the time of kidney biopsy (p = 0.000202) was the lowest, and the p value of glomerular tip adhesions (p = 0.004) was lower than the remaining 4 factors: serum albumin (p = 0.138), diastolic blood pressure (p = 0.024), global sclerosis (p = 0.019), and total glomerular adhesion (p = 0.14) (Table 1).

**Table 1:** Six clinicopathologic parameters associated with progression of IgA nephropathy identified through multiple linear regression analysis with backward elimination

	**Decrease in eGFR**	
	**Multiple linear regression**	
	**F(6.50) = 4.202565, P = 0.001692, *R***^***2***^ **= 0.335242**	
	**β**	***p *** **value**
Serum albumin	−0.23405	0.1380
Diastolic blood pressure	0.292789	0.0244
Initial eGFR	0.633544	0.0002
Global sclerosis	0.310904	0.0190
Total glomerular adhesion	−0.22385	0.1419
Glomerular tip adhesion	0.398646	0.0043

eGFR; estimated glomerular filtration rate (ml/minute per 1.73 m^2^).

Total glomerular adhesion: glomerular tip adhesion plus adhesions in other parts of the glomerulus.

### Appearance of glomerular tip adhesions

**Figure 3:**
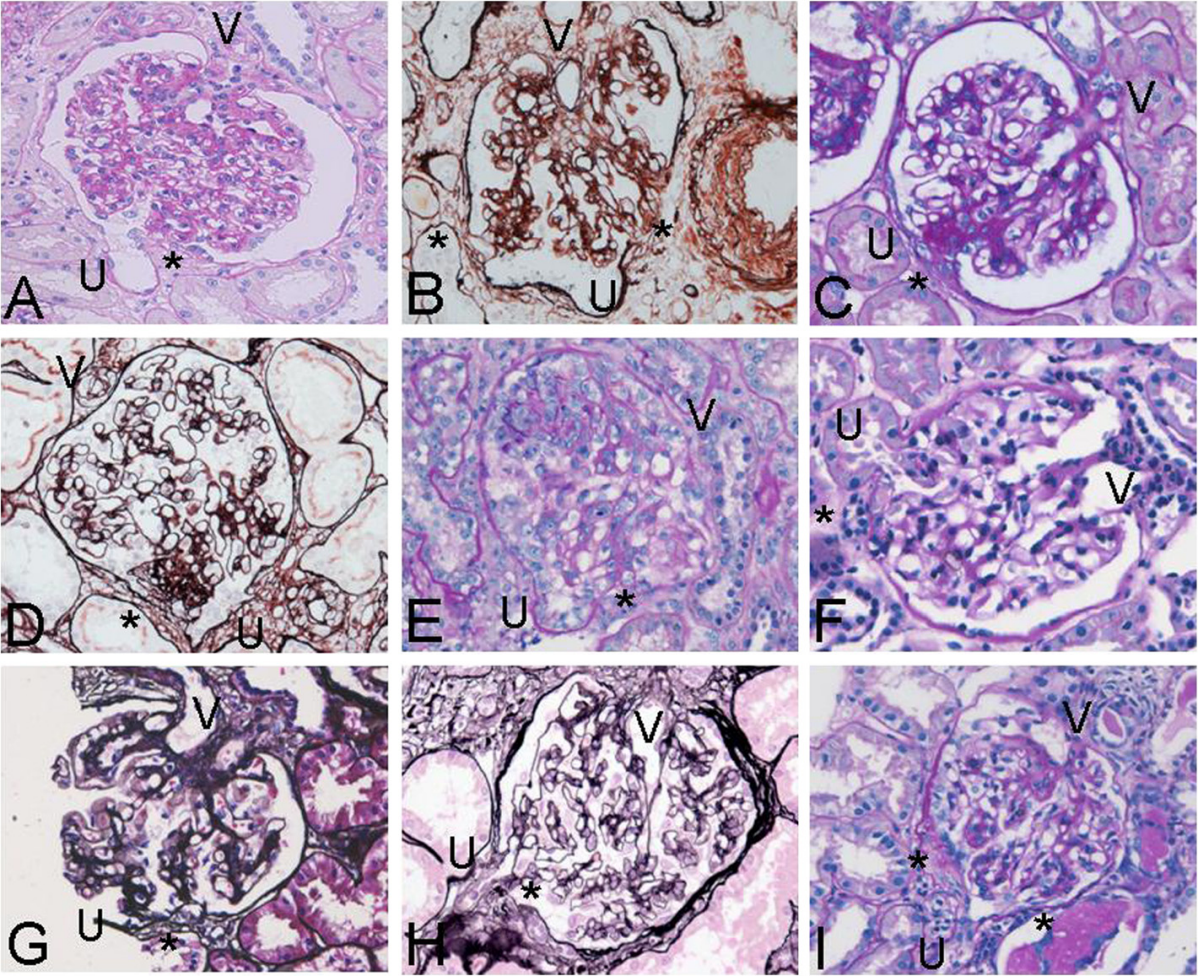
**Glomerular tip adhesions in each patient.** “V”, “U”, and “Asterisk” mean vascular pole, urinary pole, and glomerular tip adhesion, respectively. **A**. In Patient 1, a glomerular tip adhesion is seen at 6 o'clock (PAS stain, 200×). **B**. In Patient 2, glomerular tip adhesions are observed at 4 and 8 o'clock (PAMS stain, 200×). **C**. In Patient 3, a glomerular tip adhesion with segmental sclerotic lesions of the mesangial area is observed at 8 o’clock (PAS stain, 200×). **D**. In Patient 4, a glomerular tip adhesion with segmental sclerosis is present at 6 o'clock (PAMS stain, 200×). **E**. In Patient 5, a glomerular tip adhesion is seen at 6 o’clock, and hypercellularity is observed in the upper portion of the glomerulus (PAS stain, 200×). **F**. In Patient 6, a glomerular tip adhesion is observed at 9 o’clock, with mild mesangial proliferation (PAS stain, 200×). **G**. In Patient 7, a glomerular tip adhesion is seen at 7 o’clock (PAMS stain, 200×). **H**. In Patient 8, glomerular tip adhesions are observed at 7 o’clock (PAS stain, 200×). **I**. In Patient 9, glomerular tip adhesions are observed at 5 and 7 o’clock, (PAS stain, 200×).

**Figure 4:**
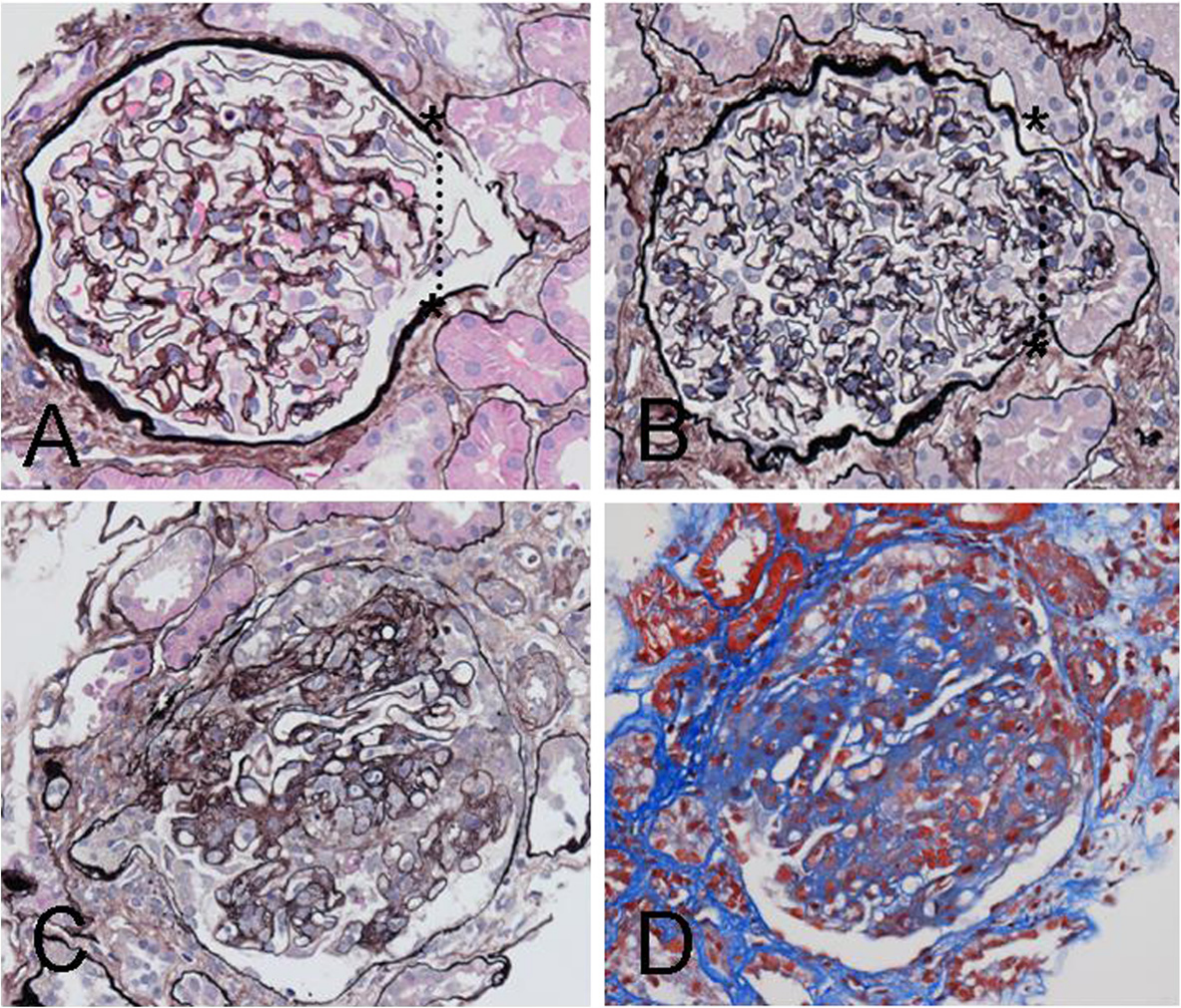
**Glomerular tip prolapse and glomerular tip adhesions accompanied by segmental sclerosis. A** and **B**. Glomerular tip prolapse, which is distinct from glomerular tip adhesions, in patients excluded from the present study. The dotted line indicates the border between the glomerulus and the proximal tubules. The glomerular component prolapses into the tubular area (PAMS stain, 200×). **C** and **D**. In this glomerulus with a glomerular tip adhesion, the upper portion shows evidence of segmental sclerosis (C: PAMS stain, 200×; D: AZAN stain, 200×).

Glomerular tip adhesions were present in 9 of 57 patients (15.8%) (Figure 2). Since 816 glomeruli were observed in these 57 patients, the prevalence of glomerular tip adhesions was 1.3% of all observed glomeruli. In these 9 patients, there were 11 out of 175 glomeruli with glomerular tip adhesions, yielding a 6.3% incidence (Table 2). Thus, the presence of glomerular tip adhesions has low sensitivity but high specificity for the progression of IgA nephropathy. Figure 3A and 3B demonstrate glomerular tip adhesions in patients without glomerular tip prolapse. Glomerular tip adhesions accompanied by segmental glomerular sclerotic changes are shown in Figure 3C and 3D. Mild mesangial proliferation was found in 4 of 9 patients with glomerular tip adhesions. Almost half of the patients with glomerular tip adhesions had evidence of segmental glomerulosclerosis, which is compatible with the pathological findings in FSGS. Figure 4 shows glomerular tip prolapse and FSGS, which differ from glomerular tip adhesions.

**Table 2:** Pathological findings in patients with glomerular tip adhesions

	**Number of observed glomeuli**	**Number of global sclerosis**	**Number of crescent formation**	**Total number of adhesion**	**Number of glomerular tip adhesion**	**Number of tip prolapse**	**Number of tip foarm cells**	**Score of interstitial damages**	**Annual decrease of eGFR**
Case 1	12	2	0	5	2	0	1	3	2.36
Case 2	18	0	0	3	2	1	0	1	2.88
Case 3	8	0	0	1	1	0	0	2	6.44
Case 4	20	8	0	5	1	0	0	3	2.92
Case 5	24	0	6	3	1	0	0	1	2.38
Case 6	15	0	8	8	1	0	0	4	3.33
Case 7	30	1	1	1	1	0	0	1	-0.51
Case 8	8	0	1	2	1	0	0	2	12.18
Case 9	40	3	0	5	1	1	0	1	5.15

### Comparison between groups with and without glomerular tip adhesions

There were no significant differences between groups with (n = 9) and without glomerular tip adhesions (n = 48) in terms of age, sex, systolic blood pressure, diastolic blood pressure, total serum protein, serum albumin, blood urea nitrogen, serum creatinine, eGFR, and proteinuria. There was a tendency towards hypercholesterolemia in the group with glomerular tip adhesions (two-sided p = 0.064; one-sided p = 0.032) (Table 3).

**Table 3:** Clinical data in patients with or without glomerular tip adhesions

	**Glomerular tip adhesion**		
	**Present**	**Absent**	***p*-value**
Number of patients	9	48	
Age (years)	38.7 ± 16.9	37.1 ± 14.6	n.s
Sex (M:F)	4:5	15:33	n.s
Systolic BP (mmHg)	134.7 ± 18.4	135.8 ± 22.4	n.s
Diastolic BP (mmHg)	82.4 ± 15.5	80.4 ± 14.1	n.s
Total protein (g/dl)	6.3 ± 0.7	6.6 ± 0.8	n.s
Albumin (g/dl)	3.9 ± 0.6	3.9 ± 0.5	n.s
Urea nitrogen (mg/dl)	15.1 ± 4.7	16.0 ± 8.5	n.s
Creatinine (mg/dl)	0.90 ± 0.27	1.02 ± 0.77	n.s
Total cholesterol (mg/dl)	230.6 ± 42.3	196.3 ± 41.7	0.064
Proteinuria (g/day)	1.39 ± 0.96	1.06 ± 1.24	n.s
Initial eGFR (ml/min/1.73 m 2)	78.9 ± 37.4	72.3 ± 32.9	n.s
Final eGFR (ml/min/1.73 m 2)	51.4 ± 38.4	62.1 ± 34.0	n.s
Observation period (days)	2894 ± 739	2768 ± 593	n.s
Annual decrease of eGFR	4.13 ± 3.58	1.49 ± 2.89	0.015

### Initial and final eGFR

**Figure 5:**
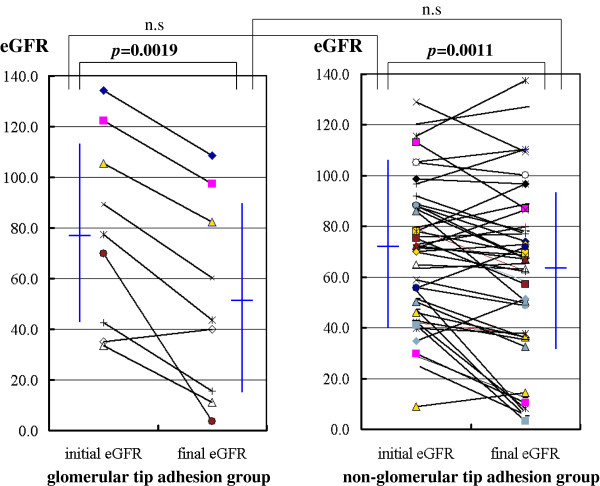
Changes in initial and final eGFR in IgA nephropathy patients with (n = 9) or without (n = 48) glomerular tip adhesions.

The initial and the final eGFR were 78.9 ± 37.4 and 51.4 ± 38.4 ml/min/1.73 m2 in the group with glomerular tip adhesions, and 72.3 ± 32.9 and 62.1 ± 34.0 ml/min/1.73 m2 in the group without glomerular tip adhesions, respectively. There were no significant differences between the two groups in terms of initial and final eGFR. However, within each group, significant differences were observed between the initial and final eGFR (p = 0.0019 and p = 0.0011, respectively) (Table 3, Figure 5).

### The annual decrease in eGFR

The annual decrease in eGFR, which are calculated as 365 (days) times ΔeGFR/observed days was 4.13 ± 3.58 in the 9 patients with glomerular tip adhesions and 1.49 ± 2.89 ml/min/1.73 m2 in the 48 patients without glomerular tip adhesions (p = 0.015) (Table 2), which means that patients with glomerular tip adhesions have a 2.77-fold more rapidly progressive course than patients without glomerular tip adhesions. This finding is reasonable, because when we first analyzed the factors predictive for a decrease in eGFR, or progression of renal function, glomerular tip adhesions were identified as one of predictive factors.

### The relationship between the glomerular tip adhesion and other pathological changes

There is no relationship between the group with and without glomerular tip adhesions with respect to the total number of glomerular adhesions and the degree of interstitial damage. In addition, there are no significant differences among patients with glomerular tip adhesions and other pathological findings. This finding means that glomerular tip adhesion is a predictive factor independent of the degree of interstitial damage (Table 1).

### The level of serum cholesterol

Serum cholesterol was 230.6 ± 42.3 mg/dl in the group with glomerular tip adhesions and 196.3 ± 41.7 mg/dl in the group without glomerular tip adhesions; this difference was significant in a one-sided test (p = 0.034) but not in a two-sided test (p = 0.064) (Table 3). This suggests that high levels of serum cholesterol or cholesterol-related substances may be associated with glomerular tip adhesions, although in theory the pathological changes do not influence serum cholesterol levels.

### The relationship between the glomerular tip adhesions and therapy

Table 4 shows the main therapies the 57 IgA nephropathy patients underwent, based on review of available data. There is no significant difference between the groups with and without glomerular tip adhesions in terms of the number of patients treated with corticosteroids only (2/9 versus 3/48), tonsillectomy only (1/9 versus 3/48), tonsillectomy plus steroid pulse therapy (3/9 versus 20/48), angiotensin-converting enzyme inhibitors (ACE-Is) (3/9 versus 10/48), angiotensin II receptor blockers (ARBs) (6/9 versus 25/48), or observation (1/9 versus 9/48). Patients receiving aggressive therapy, such as steroid pulse therapy, tonsillectomy, and tonsillectomy plus pulsed steroids, included 6 of 9 (66.7%) patients, and 26 of 48 (54.2%) patients, respectively. The type of therapy may not have been identified as a predictive factor in the present study because of the small sample size.

**Table 4:** Treatment regimens in patients with or without glomerular tip adhesions

	**Glomerular tip adhesion**	
	**Present**	**Absent**
**Therapy**	**Patient (%)**	**Patient (%)**
	9 (100)	48 (100)
Steroid only	2 (22.2)	3 (6.3)
Tonsillectomy only	1 (11.1)	3 (6.3)
Tonsillectomy plus steroid pulse	3 (33.3)	20 (41.7)
Angiotensin converting enzyme-inhibitor	3 (33.3)	10 (20.8)
Angiotensin II receptor blocker	6 (66.7)	25 (52.1)
No therapy	1 (11.1)	9 (18.8)

## Discussion

The present study demonstrates that the presence of adhesions in the glomerular tip, but not adhesions in the glomerulus overall or crescent formation, is a specific predictive factor for progression of IgA nephropathy. This is the first report regarding the importance of glomerular tip adhesions, which are associated with FSGS. The present data did not identify interstitial damage and crescent formation as predictive factors in the relatively early stages of IgA nephropathy, although these have been reported as important factors in progression.

Glomerular tip adhesions were first described as a finding in FSGS. However, the 2009 Oxford classification of IgA nephropathy [14] commented that the combined term “segmental sclerosis or adhesion” should be used, because there was no consensus on the definition of adhesions among pathologists. This international study group reported that in an analysis of 265 patients from all over the world, more than a moderate degree of mesangial proliferation, segmental glomerulosclerosis, and more than 26% interstitial damage are predictive factors for the progression of IgA nephropathy. There was no detailed information which lesion is more impact, glomerular adhesions or segmental glomerulosclerosis, because there was no discrimination of adhesions from segmental sclerosis.

Regarding FSGS, the Columbia classification defined the glomerular tip domain as the outer 25% of the tuft next to the origin of the proximal tubule [12]. Generally, glomerular tip lesions consist of tip prolapse, tip adhesion, tip sclerosis, and intracapillary foam cells in the tip domain. There are several reports about the combination of IgA nephropathy and FSGS-like lesions. In 1996, Haas [15] compared 18 of 244 (7.4%) IgA nephropathy patients with focal segmental sclerosis and capillary collapse, named as FSGS-like IgA nephropathy, with typical idiopathic FSGS patients on clinical parameters. There were no significant difference between the two groups in terms of proteinuria (5.5 ± 2.8 versus 7.7 ± 5.8 g/day), serum creatinine (1.6 ± 1.2 versus 1.9 ± 1.5), and prognosis. He commented that IgA nephropathy patients with FSGS-like lesions have a similarly poor prognosis as idiopathic FSGS patients. In 1997, Haas demonstrated that the presence of peripheral glomerular capillary deposits has no prognostic value, based on an electron microscopic study of 244 patients [16]. He did not determine the critical pathological findings such as segmental sclerosis or adhesion.

The present study points out that the presence of glomerular tip adhesions is an independent risk factor for decreasing kidney function in IgA nephropathy. The prevalence of glomerular tip adhesions, approximately 15.8% (9 of 57) of patients, and 1.3% (11 of 816) of observed glomeruli, suggests that the presence of glomerular tip adhesions has low sensitivity and high specificity for the progression of IgA nephropathy. Further analysis will reveal the importance of glomerular tip adhesions in the progression of IgA nephropathy.

**Figure 6:**
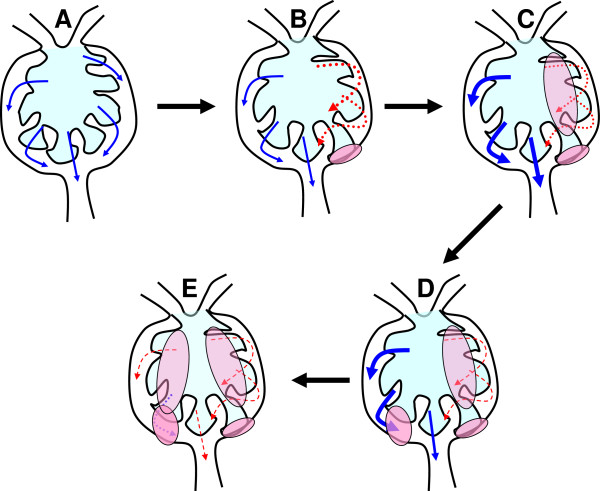
**Possible mechanism through which glomerular tip adhesions influence the progression of IgA nephropathy. A**. Intact glomerulus. **B**. A glomerular tip adhesion interferes with the flow of original urine, leading to hypofiltration on the side of glomerular tip adhesion and hyperfiltration on the intact side. **C**. Segmental glomerulosclerosis occurs due to decreased intraglomerular pressure on the side with the glomerular tip adhesion. **D**. Hyperfiltration in other areas of the glomerulus produces a new glomerular tip adhesion. **E**. The above changes finally induce global glomerular sclerosis.

Figure 6 illustrates a possible mechanism through which glomerular tip adhesions affect the progression of IgA nephropathy. Glomerular tip adhesions interfere with the flow of original urine, meaning that hypofiltration occurs on the side of the glomerular tip adhesion and hyperfiltration occurs on the intact side of the glomerulus (Figure 6A). Segmental glomerulosclerosis may develop due to the decreased intraglomerular pressure on the side of the glomerular tip adhesion (Figure 6C). Subsequently, hyperfiltration produces a new glomerular tip adhesion elsewhere in the glomerulus (Figure 6D). These changes finally induce global glomerular sclerosis (Figure 6E).

Many reports have demonstrated that young age, proteinuria, systolic blood pressure, hypoalbuminemia, initial eGFR, and severe pathological grade are predictive factors in IgA nephropathy progression [17]. The present study identified the presence of glomerular tip adhesions as an additional predictive factor. Sampling bias, resulting in 30 out of 57 patients (52.6%) with less than 1.0 g/day of urinary protein and 10 out of 57 patients (17.5%) with more than 2.0 g/day of urinary protein in the present study, may have contributed to proteinuria was not being identified as a risk factor. These results suggest that the presence of glomerular tip adhesions is a more important factor than proteinuria in the early stages of IgA nephropathy.

Hypercholesterolemia showed a weak relationship with the presence of glomerular tip adhesions (two-side p = 0.06; one-side p = 0.03), even though hypercholesterolemia was not a risk factor for the overall progression of IgA nephropathy. There are several possibilities: one is that hypercholesterolemia directly influences glomerular tip adhesions, another is that substances related to serum cholesterol induce formation of glomerular tip adhesions. Regarding the first hypothesis, lipoprotein abnormalities are a putative risk factor of the progression of chronic renal insufficiency in humans [18]. Recently, apolipoprotein L1 (APOL1) has been reported to be associated with FSGS lesions, a progressive course in HIV-related nephropathy, and chronic kidney disease [19,20]. One controversial study reported that hyperlipidemia is not an independent predictor of long-term outcome [21]. In the present study, hypercholesterolemia itself was not identified as a predictive factor for progression of IgA nephropathy. Further study is needed to clarify the relationship between APOL1 and glomerular tip adhesions. As for the latter possibility, asymmetric dimethylarginine (ADMA), an endogenous antagonist of nitric oxide (NO) biosynthesis via inhibition of the active site of NO synthase, is a strong candidate; high serum levels of ADMA have been reported to be associated with hypercholesterolemia and declining kidney function in IgA nephropathy [22]. LDL cholesterol is reported to up-regulate ADMA in endothelial cells [23,24]. Although Fuzimi-Hayashida et al. [22] found high levels of ADMA in progressive IgA nephropathy patients, unfortunately they did not comment on the relationship between ADMA and segmental sclerosis or glomerular tip adhesions. Further study is needed to elucidate the relationship between serum ADMA levels and glomerular tip adhesions.

### Limitations

The present study is limited by its single center, retrospective nature and the relatively small sample size of 57 patients, because we selected patients who could be sufficiently followed for 5 to 10 years after renal biopsy. However, the present study suggests the importance of glomerular tip adhesions, which may be related to FSGS-like lesions, in the progression of IgA nephropathy. Future studies with more patients and participating centers are needed to determine whether glomerular tip adhesions are a prognostic factor in IgA nephropathy.

## Conclusions

Glomerular tip adhesions predict decreased eGFR in relatively early stages of IgA nephropathy. High levels of serum total cholesterol may affect glomerular tip adhesions, even though hypercholesterolemia was not identified as a predictive factor for progression of IgA nephropathy.

## Competing interests

The authors declare that they have no competing interests.

## Authors’ contributions

KM corrected and analyzed data. SK performed statistical analysis. KS, WK, and SB supported correcting data. KM, NM, HM, and HI independently evaluated pathological findings. HI designed the study. All authors read and approved the final manuscript.

## Pre-publication history

The pre-publication history for this paper can be accessed here:

http://www.biomedcentral.com/1471-2369/14/272/prepub
